# Intra-operative high intensity focused ultrasound in patients with colorectal liver metastases: results of a phase I-II study in 21 patients

**DOI:** 10.1186/2050-5736-3-S1-O82

**Published:** 2015-06-30

**Authors:** David Melodelima, Aurelien Dupre, Chen Yao, Jeremy Vincenot, Jean-Yves Chapelon, Michel Rivoire

**Affiliations:** 1 Inserm, Lyon, France; 2 Centre Leon Berard, Lyon, France

## Background/introduction

Managing colorectal liver metastases (CLM) is a major clinical challenge, and surgery remains the only potentially curative treatment. Five-year survival rates of up to 51% have been recently reported. However, only 10-20% of patients are eligible for surgery, which is often precluded by the number, size and/or location of metastases, or because the necessary resection will leave insufficient volume of functional liver. A toroidal transducer for HIFU ablation has been developed and tested *in vivo* enabling destruction of large liver volumes. Preliminary *in vitro* and preclinical work demonstrated the potential, feasibility and safety of such HIFU ablations. These preclinical studies are now translated into clinical practice through controlled trials, and the aim of this study was to assess the feasibility and safety of HIFU ablation in patients undergoing hepatectomy for CLM, as well to collect preliminary efficacy and accuracy data.

## Methods

The transducer has a toroidal shape 70 mm in diameter and is divided into 32 ultrasound emitters of 0.13 cm2 operating at 3 MHz. The radius of curvature is 70 mm. A 7.5 MHz ultrasound imaging probe was placed in the centre of the device and was used to guide the treatment. The imaging plane was aligned with the HIFU focal zone. The clinical protocol was reviewed and validated by a national ethics committee according to European directives. Since this study was the first use in man of intra-operative hepatic HIFU, ablations were made only in the part of the liver scheduled for resection. This allowed real-time evaluation of HIFU ablation while protecting participating patients from any adverse effects related to this new technique. Only areas included within the planned resection were treated with HIFU. Six patients were included in the Phase I. Two single thermal ablations were created in each patient. Thirteen patients were included in Phase IIa and two HIFU ablations were to be placed precisely in a target previously identified in ultrasound images (step 1) and then at distance (step 2) from a target. Two patients were included in Phase IIb. HIFU ablations were created to ablate metastases (20 mm maximal diameter) with safety margins in all directions.

## Results and conclusions

In agreement with preclinical studies, the demarcation between ablated and non-ablated tissue was clearly apparent in ultrasound images and histology.

The dimensions measured on ultrasound imaging were correlated (r=0.88, p<0.0001) with dimensions measured during histological analysis. All HIFU ablations were obtained in 40 seconds. The average dimensions obtained from each HIFU ablation were a diameter of 21.0 ± 3.9 mm and a depth of 27.5 ± 6.0 mm. The phase IIa study showed both that the area of ablation could be precisely targeted on a previously implanted metallic mark and that ablations could be created deliberately to avoid such a mark. Ablations were achieved with a precision of 1-2 mm. In Phase IIb, one metastasis of 10 mm in diameter was ablated in 40 seconds with safety margins. Using electronic focusing a second metastasis was ablated with safety margins in 130 seconds. The dimensions of the HIFU ablation were a diameter of 48 mm and a long axis of 51 mm. HIFU was feasible, safe and effective in ablating large areas of liver scheduled for resection.

